# Examinations for the U. S. Marine Hospital Service

**Published:** 1902-06

**Authors:** Walter Wyman

**Affiliations:** Supervising Surgeon-General, U. S. Marine Hospital Service Washington, D. C.


### Examinations for the U. S. Marine Hospital Service.

*Editor Buffalo Medical Journal :*

*Sir.*—A board of officers will be convened to meet at the Marine Hospital Bureau, 3 B Street, S. E., Washington, D. C., Monday, June 16, 1902, for the purpose of examining candidates for admission to the grade of assistant surgeon in the U. S. Marine Hospital Service.

Candidates must be between 21 and 30 years of age, graduates of a reputable medical college, and must furnish testimonials from responsible persons as to character.

The following is the usual order of the examinations: (1) physical, (2) oral, (3) written, (4) clinical.

In addition to the physical examination, candidates are required to certify that they believe themselves free from any ailment which would disqualify for service in any climate.

The examinations are chiefly in writing, and begin with a short autobiography of the candidate. The remainder of the written exercise consists in examination on the various branches of medicine, surgery and hygiene.

The oral examination includes subjects of preliminary education, history, literature and natural sciences.

The clinical examination is conducted at a hospital, and when practicable, candidates are required to perform surgical operations on a cadaver.

The tenure of office is permanent. Officers traveling under orders are allowed actual expenses.

For further information, or for invitation to appear before the board of examiners, address

Walter Wyman,

Supervising Surgeon-General, U. S. Marine Hospital Service
Washington, D. C.

May 19, 1902.

